# Evolutionary Constraints to Viroid Evolution

**DOI:** 10.3390/v1020241

**Published:** 2009-09-02

**Authors:** Santiago F. Elena, Gustavo Gómez, José-Antonio Daròs

**Affiliations:** 1 Instituto de Biología Molecular y Celular de Plantas (CSIC-UPV), Campus UPV CPI access G, Ingeniero Fausto Elio s/n, 46022 Valencia, Spain; E-Mails: ggomez@ibmcp.upv.es (G.G.); jadaros@ibmcp.upv.es (J.-A.D.); 2 The Santa Fe Institute, 1399 Hyde Park Road, Santa Fe, NM 87501, USA

**Keywords:** epistasis, evolutionary constraints, genome complexity, mutation rate, RNA folding, viroid evolution

## Abstract

We suggest that viroids are trapped into adaptive peaks as the result of adaptive constraints. The first one is imposed by the necessity to fold into packed structures to escape from RNA silencing. This creates antagonistic epistases, which make future adaptive trajectories contingent upon the first mutation and slow down the rate of adaptation. This second constraint can only be surpassed by increasing genetic redundancy or by recombination. Eigen’s paradox imposes a limit to the increase in genome complexity in the absence of mechanisms reducing mutation rate. Therefore, recombination appears as the only possible route to evolutionary innovation in viroids.

## 1. Introduction

Whether evolution is erratic due to historical contingencies or it is directed along similar paths by adaptive constraints is unclear. This is a very much discussed topic among evolutionary biologists that, of course, has relevance also for our understanding of viroid evolution and diversification into the two families including several genera that have been so far recognized. Here we suggest that three different, although non-independent, sources of evolutionary constraints are operating on viroid genomes in a very intricate manner. In a first instance, RNA silencing mechanisms may impose a selective restriction that forces viroids to fold into very packed structures to escape RISC. Second, concomitant to the acquisition of a highly structured secondary RNA folding, a new constraint appears in the form of epistasis among the sites involved in the maintenance of stem-loop structures and long-distance interactions among nucleotide residues (e.g., kissing loops and pseudoknots). Particularly, when the sign of fitness effects of mutations depends on previous mutations, epistasis constrains the type and order of selected mutations, thus making future adaptive trajectories contingent particularly upon the random occurrence of the first mutation. Furthermore, if epistasis among pairs of mutations is antagonistic, then the rate of evolution dramatically slows down compared to the case of independent (or additive) mutational effects. Here, we will discuss the results of recent *in silico* analyses conducted for 29 known viroids showing that, for each case, variance exists in the sign and strength of epistasis, with antagonistic epistasis being more frequent than synergistic. Next, we will discuss the two theoretical roads to escape from the constraints imposed by antagonistic epistasis: recombination and increasing genetic redundancy. Examples of genomic duplications and recombination will be reviewed in the context of their effects on epistasis. The third source of evolutionary constraints conforms to the so-called Eigen’s paradox, by which the complexity of genomes to increase requires mechanisms that reduce mutation rates, but the evolution of such mechanisms also requires reduced mutation rates. The only available direct estimate of mutation rate for a viroid indicates that it is proportional to the inverse of genome length, as predicted by the paradox. In other words, we suggest that viroids from different genera are trapped into adaptive peaks which may not be optimal but are the results of random events in their past history, with an unexpectedly limited ability to explore sequence space simply by mutation. Given the combination of these constraints and the impossibility for increasing genetic redundancy, recombination appears as the only possible way for evolutionary innovation in the viroid’s world.

## 2. Viroids fold into compacted structures as a way to avoid RNA silencing

RNA silencing is a pan-eukaryotic, sequence-specific mechanism that plays a relevant role in controlling genome stability, development and response to biotic and abiotic stresses [1,2]. This RNA-dependent regulatory phenomenon employs double-stranded RNA (dsRNA) as cleavage precursor to generate 21- to 24-nt small RNA (sRNAs). The RNA silencing specificity is conferred by the loading of sRNAs into RNA-induced silencing complexes (RISC) that mediate the association with partially or fully complementary RNA or DNA targets [3]. In plants, in addition to its role in developmental patterning and genome stability maintenance, RNA silencing is an integral mechanism of response to adverse environmental conditions, including virus infection. As a consequence, most, if not all plant viruses have developed counter-defensive strategies to overcome the host silencing pathway. It is now well established that plant viruses encode suppressors of RNA silencing to specifically counteract the RNA silencing-based defense mechanism in order to ensure successful systemic invasion of the host plant [4].

**Figure 1.**
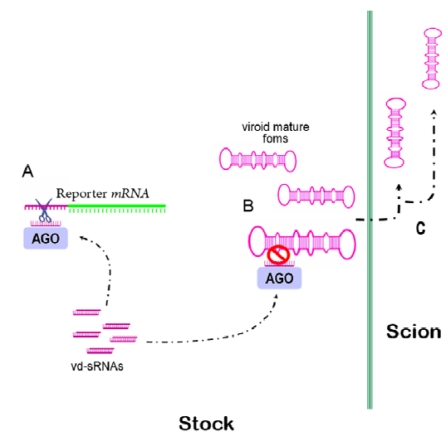
HSVd-*Nb* plants accumulate high levels of HSVd mature forms and viroid-derived (vd-siRNAs). **(a)** When these plants were agroinfiltrated with an HSVd-GFP fusion, the expression of the reporter was suppressed, indicating that vd-siRNAs are functional in guiding the specific cleavage of the full-length unstructured HSVd RNA sequence. **(b)** The resistant structured viroid mature forms evade the RNA silencing-mediated degradation and can be translocated through grafts to scions (c).

The detection of viroid-derived sRNAs (vd-sRNAs) in diverse infected hosts indicates that viroids are potential triggers of RNA silencing in infected plants. Considering that viroids do not encode any functional protein and yet are able to accumulate to high levels in infected plants, it is assumed that viroids have developed an alternative strategy to protect themselves against RNA silencing-mediated degradation. Initially, it was proposed that this protection could be conferred by a high replication rate, by compartmentalization in organelles lacking Dicer activity or by association with host proteins [5–,7]. However, a significant body of experimental evidence supports the emergent view that the highly-compact secondary structure adopted by free monomeric mature viroid forms confers significant resistance against the RNA degradation mediated by RNA silencing. This idea suggested by Wang *et al*. [8] was supported by the observation that the *Potato spindle tuber viroid* (PSTVd) replication in infected plants was resistant to RNA silencing, although viroid-specific sRNAs were active in guiding the cleavage of PSTVd-GFP fusion reporter [9]. Later, it was shown that mature forms of *Hop stunt viroid* (HSVd), resistant to RISC-mediated degradation, were able to move from cell to cell through a silenced *Nicotiana benthamiana* stock and to enter into vascular tissue for long-distance traffic to the scion in grafting assays [10] (Figure 1).

The theory that a compact RNA structure affords protection from RISC is not new since the inhibitory effect of target RNA structure on RNA silencing efficiency has been described for several animal viruses [11–13], including the demonstration that HIV-1 resists this defense mechanism by folding a target sequence into a stable RNA structure that prevents binding of specific sRNAs [14]. Indeed, a later study demonstrated that the efficiency of the RNA silencing-mediated interference showed an inverse correlation with stability of the secondary structure of the HIV-1 target sequence [15].

Consequently, viroids may have evolved a highly structured genomic RNA as a strategy to minimize the damage induced by RNA silencing-mediated degradation. This possibility is consistent with the observation that viroid variants maintain the secondary structure of wild-type strains, despite RNA sequence modification, including the insertion of additional nucleotides in their sequence [16,17]. Therefore, the question that arises is: What are the evolutionary consequences exerted by this selective pressure in viroid evolution? In the following sections we will explore the immediate evolutionary consequences of RNA folding.

## 3. A compact RNA folding means epistasis

**Figure 2.**
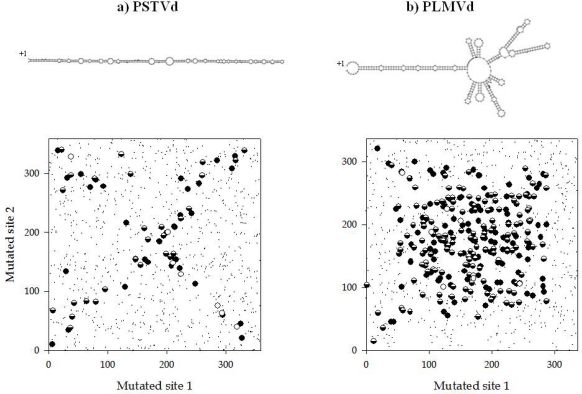
**(a)** Map of all mutation pairs (small dots) and those showing antagonistic epistasis (large dots) for PSTVd. **(b)** Same but for PLMVd. White dots indicate strictly compensatory mutations (*i.e.*, baseparing is restored in the double mutant) and fall nearly exclusively in the reverse diagonal. Semifilled dots are cases of broad-sense compensatory mutations (the effect of the double mutant on folding stability is smaller than the effect of at least one of the single mutations), whereas filled dots are noncompensatory antagonistic pairs. Taken from [20].

The way in which loci interact to produce phenotypes, *i.e.*, epistasis, has profound consequences in the evolution of organisms. Epistasis means that the fate of subsequent mutations depends upon the existence of previous mutations at other loci, therefore limiting the number of possible evolutionary pathways. In other words, epistasis creates historical contingencies. The selective accessibility of evolutionary pathways to high fitness genotypes may depend on the genetic background in which novel mutations arise. Evolutionary biologists use the term “sign epistasis” to define the situation in which the sign of the fitness effect of a mutation depends on the genetic background where it appears, i.e, beneficial in one but deleterious in another [18]. Under such conditions, a genome located at a local fitness peak may be confined to it despite the existence of higher peaks because every possible mutational pathway would create an intermediate genotype of inferior fitness. In a recent study, Sanjuán and Elena [19] explored the sign of epistasis for organisms of increasing genomic complexity, from small RNA viruses up to insects. They found an inverse correlation between epistasis and complexity: whereas antagonistic epistasis (also known as positive) was the norm for viruses, synergistic (or negative) epistasis was characteristic of eukaryotic organisms, with bacteria occupying an intermediate position characterized by average additive (no epistatic) interactions among loci. This relationship being true, then it is reasonable to expect viroid genomes as being characterized by an abundance of antagonistic epistases. This expectation was tested by Sanjuán *et al.* by using a computational approach [20]. The effect of all possible pairs of mutations on the stability of the predicted secondary structure was estimated for 29 viroids. For each possible pair, an epistasis coefficient was computed and the resulting value classified into 3 categories depending on whether they interacted additively, synergistically or antagonistically [20]. Additivity of mutational effects was the rule [20]. However, when a pair of mutations was involved in a nonadditive way, antagonistic epistases were significantly more abundant than synergistic epistases for all viroids except for *Citrus bent leaf viroid* (CBLVd), for which both types of epistases were equally common. Next, these authors sought to explore whether the taxonomic relationships between viroid families influenced the extent and sign of epistasis. Indeed, the analyses showed that the amount of epistatic interactions significantly increased with the number of hairpin loops, being more abundant in the highly branched structure of *Pelamoviroid* (family *Avsunviroidae*) than in the rod-like structure of *Pospiviroidae* [20]. Figure 2 illustrates this finding for two representative members of these families, *Peach latent mosaic viroid* (PLMVd) and PSTVd, respectively. For PSTVd, at best, a single structural domain can be defined, the entire rod. As it can be seen in Figure 2a, positions showing antagonistic epistasis lie along the 2 diagonals, suggesting that, in general, antagonistic epistasis appears as a consequence of maintaining the rod-like structure (e.g., among compensatory mutations indicated as semi-filled dots in Figure 2) but also by multiple mutations hitting the same secondary structure element (filled dots in Figure 2). By contrast, the distribution of epistatic pairs turned out to be more complex for the highly branched PLMVd (Figure 2b), and the above pattern can only be observed for the largest left stem of the structure.

There are two direct evolutionary consequence of an excess of antagonistic epistasis. The first one is a reduction in the rate of adaptive evolution [21]. The reason for this slowdown is as follows. If two sites are paired in the secondary structure and involved in an antagonistic interaction, replacing one of them will affect the correct folding, uncovering a deleterious fitness effect that will be larger than expected if sites interacted in an additive way thereby creating a genotype with a fitness lower than either the wildtype or the double mutant. Therefore, this intermediate form will be quickly displaced from the population by the wildtype. Consequently, the double mutant can only be directly produced by a double mutation event, a much more unlikely process than the gradual accumulation of single mutations by natural selection. The second evolutionary consequence is that the limitation in the number of viable adaptive paths almost ensures that independently evolved lineages will converge into the same evolutionary solution. Indeed, viroid convergent evolution has been described in several cases. In one of the most illustrative examples, a recent report showed that independently evolved lineages of *Citrus exocortis viroid* (CEVd) maintained for several years in either trifoliate orange or sour orange trees converged into a host-specific genotype [22]. Furthermore, when the evolved CEVd population was re-inoculated onto the ancestral Etrog citrus host we recovered the same ancestral master sequences [22], a result that further stresses the existence of severe evolutionary constraints.

### 3.1. Two ways to escape from the tyranny of antagonistic epistasis: genetic redundancy and recombination

Can a genome escape from the evolutionary constraints imposed by antagonistic epistasis? The answer is yes. Two possible mechanisms can be invoked to this end: genetic redundancy and recombination.

Firstly, in theory, the existence of genetic redundancy relaxes antagonistic epistasis [19,23–26] because it increases the chances for mutations to reduce their impact in fitness (in the extreme case becoming neutral) and, thus, increasing additivity. Sanjuán *et al*. [20] tested this prediction taking advantage of the existence of 3 viroid species for which natural variants with partial genome duplications have been identified. First, it is important to notice that all duplicated segments maintain the perfect secondary folding, suggesting that strong selection exists to avoid unfolding regions in the molecule. CEVd variant D-104 contains a duplication of 104 nt that affects the right terminal domain of the folded molecule compared to the CEVd-C isolate [27].

**Figure 3.**
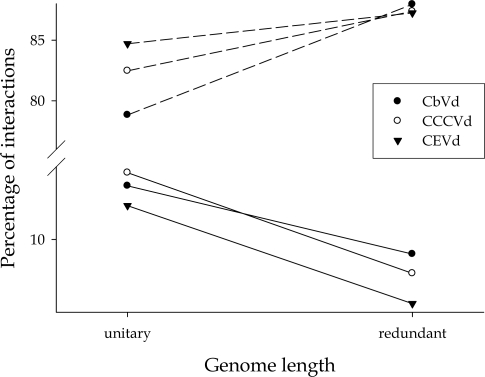
Changes in the fraction of mutation pairs with additive (dashed lines) and antagonistic (continuous lines) effects as a consequence of increasing genome complexity for CEVd C, CCCVd fast and CbVd-1. Taken from [20].

Similarly, the slow variant of *Coconut cadang-cadang viroid* (CCCVd) possesses a duplication of 41 nt at the right terminal domain compared to the CCCVd-fast isolate [28]. The case of *Coleus blumei viroid* types 1 (CbVd-1) and 3 (CbVd-3) is somehow more complex: although it does not represent a clear case of genomic duplication, as in the previous two examples, it certainly represents an instance of additional genetic material incorporated in the genome. CbVd-3 is 116 nt longer than CbVd-1 but this difference in length results from the insertion of short stretches of nucleotides in both the upper and lower strands of the rod-like structure plus the addition of terminal loops in the CbVd-1 genome [29]. To test this prediction, all pairs of random mutations were obtained for the standard sequence and the “redundant” one and the amount and sign of epistasis compared among them. Figure 3 illustrates the results obtained. First, the result is consistent for all three pairs of viroids, showing that the number of antagonistic interactions significantly decreases as genetic redundancy increases. Concomitantly, the number of additive interactions increases in a similar magnitude. Therefore, increasing genomic complexity contributes to reducing the amount of antagonistic epistasis.

Secondly, recombination has also been theoretically claimed as a mechanism for reducing antagonistic epistasis [30]. In theory, sex favors the generation of longer and more modular genomes with relaxed antagonism [31]. Then the question that needs to be answered is how often recombination occurs during viroid evolution. Although an exhaustive analysis of viroid recombination has not yet been undertaken, several viroids have been dubbed as chimeras. The evidence goes back in time to the characterization of *Tomato apical stunt viroid* (TASVd) and *Tomato planta macho viroid* (TPMVd) as putative recombinants containing sequences from PSTVd and CEVd [32]; all these viroids belong to the genus *Pospiviroid*. Recombinant viroids containing fragments from two different genera within the family *Pospiviroidae* have also been described, such as (i) *Columnea latent viroid* (CLVd) whose sequence resembles other members of the genus *Pospiviroid* but contains a CCR highly similar to that of HSVd (the only member of the *Hostuviroid* genus) [33], (ii) *Citrus viroid* type IV (CVd-IV), a *Cocadviroid* proposed to be the result of a recombination event between a *Pospiviroid* (most likely CEVd) and HSVd [34], and (iii) *Australian grapevine viroid* (AGVd), an *Apscaviroid* with strong similarities with the *Pospiviroid* CEVd [35]. The molecular mechanism by which recombinant viroids can be produced during coinfection of a common host involves discontinuous transcription by an RNA polymerase that jumps between templates [32]. In addition to all these recombination events involving viroids from the same or different genera, the above mentioned enlarged variants containing partial genomic duplications also represent a different case for recombination. Interestingly, recombination has never been reported between members of the family *Avsunviroidae*.

**Figure 4.**
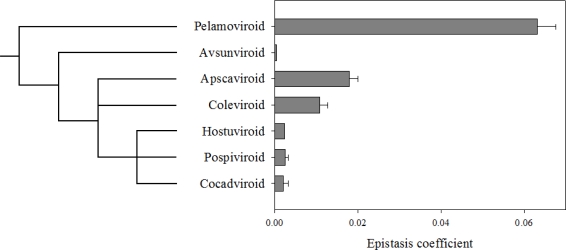
Distribution of epistasis coefficients over the viroid taxonomy. The phylogenetic tree shown has been adopted from [36]. The root of the tree was placed by comparing the viroid sequences with those from the viroid-like RNA satellites. Taken from [20].

Sanjuán *et al*. [20] also evaluated whether an evolutionary trend in the sign of epistasis existed. Figure 4 shows the average epistasis for 7 viroid genera. The phylogenetic tree was rooted using the viroid-like satellite RNAs as the outgroup [36], with which the viroids share certain structural properties [36,37]. According to this tree, the autocatalytic viroids (*Pelamoviroid* and *Avsunviroid*) represent more ancient lineages than the *Hostuviroid*, *Pospiviroid* and *Cocadviroid*. As illustrated in Figure 4, a negative correlation exists between the magnitude of antagonistic epistasis and the phylogenetic depth (*i.e.*, a measure of ancestry), with the exception of *Avocado sunblotch viroid* (ASBVd), which shows much weaker antagonistic epistasis that would be expected by just looking at its phylogenetic location. This correlation suggests that during their evolutionary radiation, viroids have changed their genomic architecture from one characterized by strong antagonistic epistasis, as a consequence of multi-branched folding of the mature forms, to another one characterized by weaker antagonism characteristic of the rod-like conformation. The fact that recombination seems to be common among *Pospiviroidae* but not among *Avsunviroidae* further reinforces the differences in the amount of antagonistic epistasis among these two families.

If the necessity for evading the RNA silencing machinery imposes a strong selective constraint favoring a highly folded structure in the mature form of viroids, how can we explain this evolutionary trend towards reducing antagonistic epistasis? First, it has to be stressed that even for the rod-like viroids epistasis is, on average, significantly antagonistic, thus suggesting that the selective pressure is weak. Second, perhaps a tension exists between the necessity of a strong folding and other important fitness traits. The question is then if we can identify this potential second fitness trait. We hypothesize that structural robustness to mutational effects represents a good candidate. A causal connection exists between epistasis and robustness, and reduced antagonistic epistasis can evolve if selection favors robustness [38,39]. The exceptionally high mutation rates for viroids (see below) create the conditions in which selection favors robustness [40,41]. Indeed, Sanjuán *et al*. [42] have shown that viroids may have been experiencing an evolutionary trend towards increasing structural robustness paralleling the reduction in antagonistic epistasis illustrated in Figure 4. In the case of *Avsunviroidae*, the presence of the hammerhead imposes important structural constraints, likely linked with the branched structure, and the nucleotide sites involved in correctly establishing the hammerhead are also involved in other structural elements [43]. Therefore, mutations may have strong pleiotropic effects beyond altering the local stem-loop structure in which they appear and have strong deleterious fitness effects. *Pospiviroidae* would have relaxed the structural constraints and evolved towards rod-like forms which are more robust against deleterious mutations affecting secondary structure [42] (*i.e.*, mutations will have weaker fitness effects) and characterized by weaker antagonism [20].

## 4. Small genomes and high mutation rates: the Eigen’s paradox

Except for the possible role of secondary structures, factors determining the mutation rate of viroids escape their own control and hence, the evolution of mutation rates should be mainly governed by thermodynamic noise and host factors. Viroids are transcribed by host DNA-dependent RNA polymerases (DdRp) using RNA as a non-natural template. Many studies have shown that after inoculation of plants with viroid cDNAs or transcripts, polymorphic populations are quickly generated (this topic has been recently reviewed in [42]). An important and well confirmed observation is that the degree of genetic variability varies across families: in standardized inoculation experiments, the members of the family *Avsunviroidae* show more haplotypes and more nucleotide differences among haplotypes within an infected plant than members of the *Pospiviroidae* [43]. However, this observation does not necessarily mean that the former have a higher mutation rate than the later. Indeed, the frequency of mutant genotypes in a population also depends on the fitness effects of different mutations and on the strength of genetic drift. Durán-Vila *et al*. undertook an *in silico* approach to estimate the mutation rate of viroids [43]. These authors estimated the genomic mutation rate of the *Avsunviroidae* to be 10-fold larger than that for the *Pospiviroidae* and suggested that this difference would be due to two factors: (1) while the *Pospiviroidae* are transcribed by the multimeric RNA polymerase II [44], the *Avsunviroidae* would be replicated by a nuclear-encoded chloroplastic DdRp which is structurally similar to the monomeric polymerases of bacteriophages [45]; (2) the chloroplast may represent a more mutagenic environment than the nucleus as a side effect of the electron transduction during photosynthesis that produces free radicals. In a recent study, Gago *et al*. [46] used the frequency of lethal mutations generated *de novo* in a replicating population to estimate for the first time the *in vivo* mutation rate of the *Avsunviroidae* member *Chrysanthemum chlorotic mottle viroid* (CChMVd). The obtained figure was 0.0025 mutations per site and replication round, which translates into a genomic mutation rate of 399 × 0.0025 ≈ 1 mutations per genome and replication round. Assuming that the theoretical computations described in [43] are correct, this would imply that for the *Pospiviroidae* the genomic mutation rate would be one order of magnitude lower, or 0.1.

M. Eigen hypothesized that replication fidelity defines a limit to the amount of genetic information that can be encoded in a genome without incurring an excessive mutational load that would jeopardize the integrity of the encoded message [47]. Genome complexity can only increase if replicases become more complex by incorporating mechanisms for reducing mutation rate but, in a clear chicken-or-egg paradox, these more complex polymerases would themselves require a lower mutation rate to ensure integrity. Indeed, according to Eigen’s computations, the maximum genome size would scale as the inverse of the mutation rate. The above estimate of mutation rate for CChMVd matches this expectation extremely well (1/0.0025 = 400 versus the observed 399 nucleotides), indicating that CChMVd replicates at the maximum possible mutation rate compatible with the size of its genome. However, extending this argument to the case of the *Pospiviroidae*, the 10-fold lower mutation rate estimated in [43] would clearly be an underestimate since genome lengths for the pospiviroids (from 246 nt for CCCVd to 371 nt for CEVd) are in the same range as those for *Avsunviroidae* (from 247 nt for ASBVd to 399 for CChMVd, the largest known viroid). This discrepancy would be solved when experiments as those described in [46] are performed for a pospiviroid.

In general terms, a parasitic lifestyle may also favor faster replication as a way to avoid host defenses. If a tradeoff exists between replication fidelity and efficiency, an increase in fidelity will come at a cost in efficiency either because of the extra time required for replicating the additional genetic material necessary for fidelity or because of the slowdown in the replication process associated with error detection and correction [48,49**Error! Reference source not found.**]. Furthermore, an increase in fidelity may be detrimental in the long run since it may reduce the likelihood of producing beneficial mutations, although this cost may be compensated in the short run by reducing deleterious mutational load [50,51]. In such circumstances, a parasite may opt for an efficient but low fidelity replication. The necessity for fast replication together with Eigen’s paradox constrains the maximal length the genome of a viroid can reach.

## 5. Conclusions

The necessity to avoid the first line of plant defense, RNA silencing in the absence of suppressor proteins, may have forced viroids to adopt an escape strategy based on a highly compact secondary structure. However, this compact structure has an unavoidable consequence of tremendous evolutionary impact: it creates antagonistic epistasis among nucleotide sites. Antagonistic epistasis limits the number of alternative evolutionary pathways, slows down the rate of adaptation and retains genomes stuck in local adaptive peaks despite the existence of higher peaks in the neighborhood of sequence space. The only two ways to escape from the tyranny of antagonistic epistasis are (i) to increase genetic redundancy by duplicating functional parts and (ii) to increase genetic diversity by recombination that generates modular genomes and promotes long-distance jumps over sequence space. Duplications have been described for a few *Pospiviroidae* but, so far, are of unclear biological relevance, and their effects on fitness have not been properly evaluated *in vivo*. Furthermore, longer genomes face a second constraint: very high mutation rates are incompatible with longer genomes. In other words, to generate a viroid genome able to escape from the constraint of antagonistic epistasis would require reducing mutation rate well below the inverse of the genome length. Unfortunately for viroids, they do not control their mutation rate and entirely rely on cellular DdRps operating on an RNA template. Therefore, recombination appears as the only way by which viroids may evolve novelty and explore sequence space. Indeed, as we have reviewed here, recombination has played an important role in the genesis of new viroid species within the *Pospiviroidae* but has not been described yet among the *Avsunviroidae*.

All our above presentation builds upon the hypothesis that RNA silencing forces viroid molecules to be folded in a compact way. However, this is clearly not the only source of evolutionary constraint. Although we have not elaborated on the role that functionally important RNA structures may play on viroid evolution, it should be clear that the necessity of preserving structures, such as the hammerhead ribozyme involved in the processing of the oligomeric forms produced during *Avsunviroidae* replication [52] or the loop motifs present in PSTVd that are associated with replication and trafficking [53], will create additional evolutionary constraints which in some cases may also produce antagonistic epistasis.
